# Advancements in Oral Delivery Systems for Probiotics Based on Polysaccharides

**DOI:** 10.3390/polym17020144

**Published:** 2025-01-08

**Authors:** Zi-Dan Wang, Wei Zhang, Tian-Xin Liang

**Affiliations:** 1 College of Chemical and Biological Engineering, Zhejiang University, Hangzhou 310058, China; wangzidanly@126.com (Z.-D.W.); zhangw@eastchinapharm.com (W.Z.); 2 Hangzhou VicrobX Biotech Co., Ltd., No. 700 Shixiang Road, Hangzhou 310015, China

**Keywords:** probiotics, polysaccharide, technology, oral delivery system, microparticle

## Abstract

Probiotics are an essential dietary supplement for intestinal flora balance, inhibition of pathogenic bacteria and immune regulation. However, probiotic inactivation during gastrointestinal transportation remains a big challenge for oral administration. Hence, oral delivery systems (ODSs) based on polysaccharides have been constructed to protect probiotics from harsh environments. Cellulose, chitosan, alginate and their derivates have been used to form a protective layer for probiotics. This review summarizes the superiority and application of polysaccharides in forming protective layers for probiotics. Meanwhile, ODS processes including extrusion, emulsion and spray drying are also summarized. The preparation technique mechanism, the microparticle formation process and especially the role polysaccharides serve in the preparation process are overviewed. Lastly, the need for cell viability retention during the dehydration and construction of core-shell ODS microparticles is emphasized in this review.

## 1. Introduction

Probiotics were defined as “live microorganisms which, when administered in adequate amounts, confer a health benefit on the host” by the Food and Agriculture Organization (FAO), United Nations (UN) and World Health Organization (WHO) formally in 2001 [1,2]. Probiotics encompass a wide variety, with the most well-known types primarily comprising *Bifidobacterium*, *Lactobacillus*, *Lacticaseibacillus*, *Streptococcus* and *Lactococcus*, among others. A multitude of research has reported on the physiological benefits achieved by probiotics [2,3]. Probiotics have been proven to not only improve general health conditions such as lactose intolerance and constipation but also have specific beneficial effects on certain disease states. These include conditions such as irritable bowel syndrome (IBS), obesity, atopic dermatitis, type II diabetes (T2D), Alzheimer’s disease and various types of cancer [4,5,6,7,8,9,10,11]. Diverse probiotic strains confer health benefits to humans through distinct mechanisms [5] The complex interplay of these mechanisms can be broadly categorized into critical areas, which include but are not limited to the modulation of gut microbiota [12], improvement in gut barrier function, prevention of pathogenic translocation, elevation of beneficial short-chain fatty acids, regulation of aquaporin protein expression for improved hydration [13], adjustment of neurotransmitter levels for enhanced nervous system signaling [14] and reduction in inflammation [15]

Due to the aforementioned benefits, probiotics have become increasingly popular in recent years. Probiotics are now widely used in the fields of functional foods, dietary supplements, pharmaceuticals, agriculture and pet care. However, they still face significant challenges in their practical industrial applications. Given that probiotics display notable sensitivity to external environmental parameters such as temperature, humidity and oxygen, careful consideration must be given to their handling and processing [16]. Additionally, they are also susceptible to the internal elements encountered in the human body, including harsh conditions like stomach acids, bile salts and digestive enzymes. Thus, despite their high initial numbers, the actual viable, effective probiotics reaching the gut may be significantly reduced. Ensuring maximum ex vivo stability of probiotics throughout preparation, storage and processing, while also securing high survival rates in the intestinal environment post-consumption, poses a formidable challenge in the manufacture of probiotic formulations. To address these challenges, efforts are devoted to improving encapsulation technologies with the aim of ensuring probiotics’ longevity and functional capabilities. These techniques are meant to safeguard probiotics during their shelf life, ensuring their vitality so they can quickly “awaken” once they reach the gastrointestinal tract. This is to assure maximum probiotic survival and efficacy upon entering the gut environment. Designing efficient encapsulation techniques to sustain probiotics’ viability and guarantee their prompt revival represents a pressing technological challenge that requires innovative and groundbreaking solutions [16].

Various polymers have been applied for encapsulating probiotics to improve their stability in gastrointestinal environments. Among the delivery system backbones, polysaccharides demonstrate superiority in biocompatibility, biodegradability and mucosal adhesion [17]. Polysaccharides are composed of monosaccharides connected by glycosidic bonds. The abundant hydroxyl and charged groups endow polysaccharides with potential in inherent gelling properties, crosslinking by ionic agents, resistance to acid degradation and colon-specific degradation, which overcome the limitations of the oral delivery of probiotics [18,19].

Although a summary of biopolymer-based ODS probiotics has been reported, key considerations throughout the design of ODSs are still necessary to be analyzed. This research emphasizes the advantages and modification strategies of polysaccharides in the field of probiotics oral delivery systems (ODSs). Meanwhile, preparation techniques applied for polysaccharide-based ODSs including extrusion, emulsion and spray drying, their technical principles, and their superiority are summarized. Moreover, the restrictions of ODSs and future perspectives focused on cell viability retention during dehydration and core-shell microparticle construction are proposed, which aim to provide new insight into precise probiotic delivery.

## 2. Polysaccharides for Probiotic Encapsulation

The oral administration of probiotics has been restricted in its therapeutic effect as a result of cell inactivation during gastrointestinal transportation. Although various synthesized polymers such as Eudragit L 100 have been applied to encapsulate probiotics to achieve responsive release in the colon, polysaccharides show superiority in their ease of access, abundant side chains, biocompatibility and biodegradability [20,21]. Moreover, a polysaccharide-based matrix reveals unique advantages in achieving multiple responsive mechanisms as well as enhancing mucus adhesion, which are essential for precise delivery and prolonging retention time in the colon [22]. A sufficient adhesion duration is a prerequisite for cell proliferation and flora regulation.

### 2.1. Cellulose

Cellulose is a liner polysaccharide composed of glucopyranose monosaccharides linked by β-1,4-glucosidic bonds [23]. Natural cellulose is insoluble in the digestive tract, which is beneficial for maintaining the mechanical strength of encapsulation systems and preventing the penetration of gastric fluids during digestive tract transportation [24]. Nanocrystal cellulose, nano-sized crystal extracted from natural fiber, has been applied as a picker emulsion stabilizer owing to its low density and interfacial adsorbability. Meanwhile, the abundant glucuronyl and hydroxyl groups endow cellulose with a negative charge, which is essential for the repulsion between droplets and the stability of probiotic-loaded emulsion [25]. In addition to natural cellulose, cellulose acetate is a cellulose derivate prepared by substituting cellulose hydroxyl with acetyl groups. The strong mechanical strength of cellulose acetate makes it suitable for the preparation of probiotic electrospinning. Free *Escherichia coli* Nissle 1917 demonstrated intolerance to simulated gastric fluids (SGFs) while decoration with cellulose acetate-based composites significantly inhibited cell inactivation with a slight decrease by 5.0 log CFU/mL after incubation in SGF for 2 h [26].

In order to broaden their application in aqueous systems, water-soluble cellulose derivates such as sodium carboxymethylcellulose (CMC-Na) and cellulose sulfate were synthesized. CMC-Na shows resistance to a gastric acid environment and favorable solubility in the intestine, which makes CMC-Na an ideal matrix for oral probiotic delivery [27]. Cellulose sulfate is another water-soluble cellulose derivate. A sufficient amount of sulfate groups is able to buffer the H^+^ ions of stomach fluids and provide prevention for probiotics [28]. Gunzburg et al. reported that cellulose sulphate capsules effectually protected *Lactobacillus casei* DSM 20011 from the corrosion of SGF with pepsin (SGFP) and lysozyme. In contrast, the viability of cells without encapsulation decreased by 8 logs by exposure in SGFP for 1 h [29].

In addition to natural cellulose and its derivates, bacterial cellulose produced by bacteria arouses wide attention due to its unique fiber structure, water retention capacity and high degree of polymerization [30,31]. Fijalkowski et al. presented a novel bacterial immobilization strategy by co-incubation of *Lactobacillus delbrueckii* PKM 490 with *Gluconacetobacter xylinus* DSM 46604, which has been applied for cellulose synthesis. Bacterial cellulose secreted by *Gluconacetobacter xylinus* DSM 46604 formed an intact bead to embed *Lactobacillus delbrueckii* PKM 490 in the inner matrix during incubation. The immobilization provided a secure shelter for living cells from the harsh digestive environment [32] The application of cellulose and their derivates in probiotic encapsulation has been listed in Table 1.

### 2.2. Chitosan

**Figure 1:**
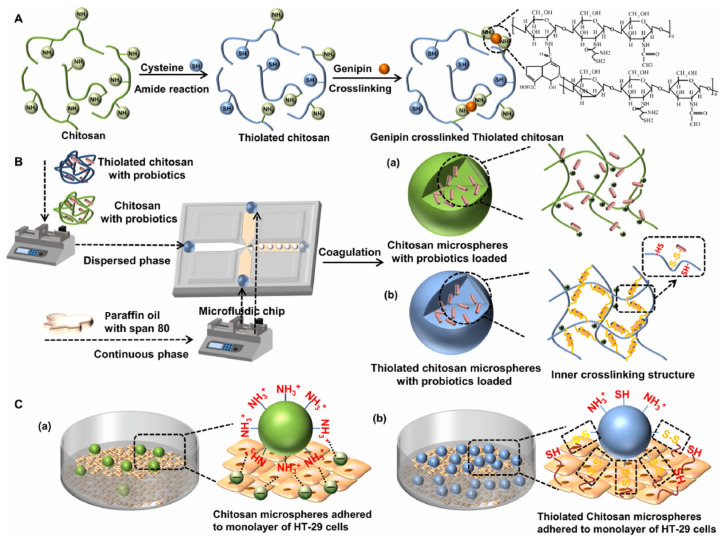
Mucus adhesion mechanism of chitosan and thiolated chitosan microparticles. (**A**) Synthesis route of crosslinked thiolated chitosan; (**B**) Chitosan or thiolated chitosan microspheres loaded probiotics; (**C**) mucus-adhesion mechanism of chitosan or thiolated chitosan microspheres [40].

Chitosan, a deacetylated product of chitin, has emerged as an ideal polymer for oral delivery systems [36]. In addition to its gastric resistance due to its protonation and deprotonation of functional groups, chitosan also exhibits great mucus adhesion which prolongs its probiotic localization in the gut [37]. Chitosan is composed of β-(1–4)-linked anhydro-D-glucosamine and anhydro-N-acetylglucosamine. Its protonation of amino groups endows chitosan with a positive charge and prompts its electrostatic interaction with negatively charged glycoproteins [38]. In addition, the mobility of a chitosan chain is conducive to twining with mucus protein to form chain entanglements and enhance mucus adhesion [39]. Recently, modified chitosan with cysteine has been verified to improve mucus adhesion. As shown in Figure 1, a thiol group grafted to chitosan could crosslink with mucus by forming disulfide bonds, leading to increased mucus adhesion, which was 2.27 times higher than that of unmodified chitosan [40]. Enhanced mucus adhesion is beneficial for prolonging probiotic residence time, thereby improving the therapeutic effect of probiotics.

Chitosan not only possesses gastrointestinal resistance and mucus adhesion, but also acts as an inhibitor of inflammation. Chitosan has been verified to inhibit inflammatory responses caused by lipopolysaccharides, indicating its potential in oral encapsulation systems for the treatment of inflammatory bowel disease [41]. Therefore, probiotic-loaded chitosan vehicles could also serve therapeutic and regulatory functions.

### 2.3. Alginate

Alginate is a linear polysaccharide composed of randomly arranged D-mannuronate and α-*L*-guluronate. Known for its gel-forming property, alginate is widely applied as a coating polymer for oral delivery systems [42]. A typical crosslinking network of alginate-based gel can be developed by ionic interaction.

**Figure 2:**
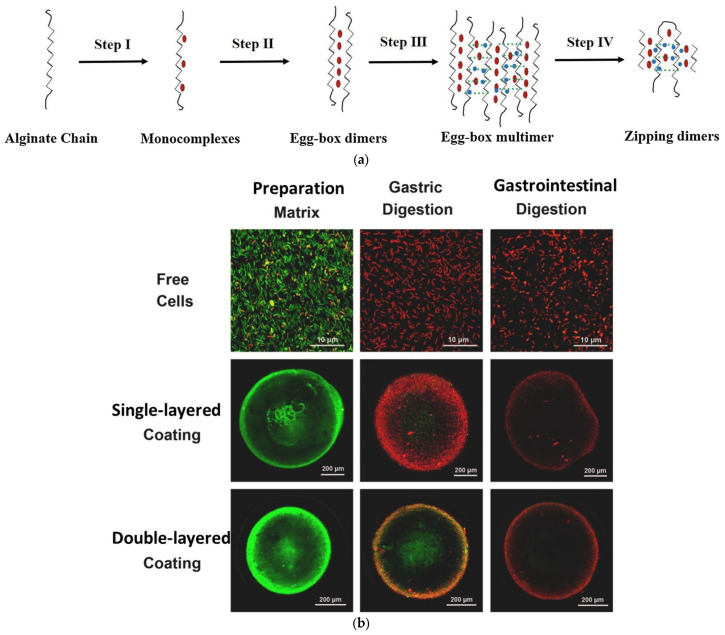

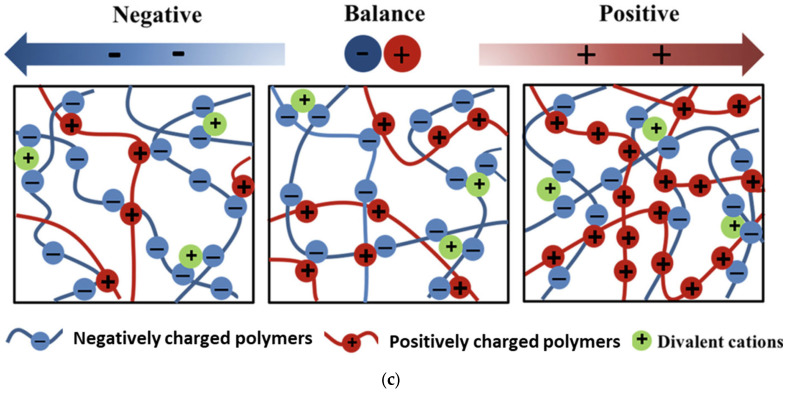
Crosslinking mechanism and oral delivery superiority of alginate-based encapsulation systems. (**a**) “Egg-box” structure of alginate- Ca^2+^ crosslinking network [47]. (**b**) Double-layered calcium alginate coating beads showed an improved proteiction to *L. acidophilus* in GI envrironment. Green fluorescence represents live cells while damaged cells emitted red fluorescence [45]. (**c**) Crosslinking structure of polycation/lginate polyelectrolyte complex [46].

As shown in Figure 2a, the addition of Ca^2+^ can induce guluronic acid (G) to bind with adjacent G residues and form an alignment conformation. The hydrophilic cavities of G-G chains tend to coordinate with Ca^2+^, resulting in an “egg-box” structure of the ionic alginate network [43]. As a coating layer for probiotic-loaded systems, the protonation of alginate residues into –COOH prevents simulated gastric fluid (SGF) penetration and probiotic degradation in acidic gastric environments, while deprotonation leads to the disaggregation of entanglements and pH responsiveness. An alginate-Ca^2+^-based coating layer was developed to embed *Escherichia coli* Nissle 19175 and 5-aminosalicylic acid for the synergistic therapy of intestinal diseases, which indicated potential in precise synergistic therapy for colitis, owing to the effective retainment of cell viability and pH-responsive erosion at the focal site [44]. Mirmazloum et al. constructed double-layered calcium alginate beads to preserve *L. acidophilus* viability in the gastrointestinal tract. It can be seen from Figure 2b that multilayered encapsulation is an effective strategy for maintaining cell viability during gastrointestinal (GI) digestion [45]. However, the single ionic alginate layer is susceptible to SGF due to its incompact and porous structure. In order to obtain a dense coating layer, polycations cooperate with alginate (Figure 2c). Different from ionic crosslinking, interactions between polycations and alginate consist not just of electrostatic attraction, but also of hydrogen bonding and van der Waals forces, demonstrating potential in reliable resistance during gastrointestinal transportation [46].

### 2.4. Starch

Natural starch is unstable in GI environment due to enzyme degradation which limits its application in ODSs. Different from natural starch, resistant starch is a kind of dietary fiber that is tolerant against enzymatic hydrolysis in the small intestine, while it can be degraded in the colon, endowing it with potential in its application as a probiotic carrier. Water chestnut starch-based nanoparticles were prepared to enhance the stability of *Lactobacillus casei* MTCC 297 in the gastrointestinal tract. An improvement of cell viability from 4.87 to 8.87 CFU/mL was observed by water chestnut starch embedment [48].

Chemical modification is an efficient strategy for obtaining starch derivates with anticipative characteristics. Considering that structural properties may have an influence on encapsulation efficiency and protection ability, Cortes et al. synthesized phosphorylated, acetylated and succinylated starches to compare their functional diversity. Compared with other modified starches, succinylated starch maintained the best cell viability of *Bifidobacterium breve* ATCC 15700 during storage [49]. In addition to side chain modifications, crosslinking is also a common method for obtaining satisfactory encapsulation material. Ashwar et al. reported a phosphorylation-resistant starch with a crosslinking structure that encapsulate probiotics by porous adsorption. As a kind of potential prebiotic, resistant starch provided satisfactory protection to *Lactobacillus brevis* MTCC 01, *Lactobacillus casei* MTCC 297 and *Lactobacillus plantarum* MTCC 021 during storage. Its survival rate was also significantly enhanced in SGF and simulated intestinal fluid (SIF) [50].

### 2.5. Gum Arabic

Gum arabic (GA), composed of glucuronic acid, arabinose, galactose and rhamnose, is a kind of polysaccharide with high water solubility. GA exhibits satisfactory film-forming ability as well as intestinal bacterial proliferation regulation function, making it an ideal encapsulation matrix for probiotic ODSs [51]. GA has been applied in different dosage forms such as in solid lipid microparticles, nanofibers, emulsions and microcapsules [51,52,53]. *Lactobacillus plantarum* NRRL B-4496 was embedded in solid lipid microparticles with GA as the matrix. Compared with whey protein isolate, GA was demonstrated as providing a better shelter for probiotics from the outer environment, which prevented cell inactivation during GI transportation [54]. In addition, GA also acts as a potential electrospun material. Fareed et al. reported that GA was miscible with polyvinyl alcohol (PVA) regardless of its semi-crystalline state. Prepared nanofibers revealed its mechanical stability and reliable protection of probiotics [55].

## 3. Preparation Technique for Probiotic ODSs

A microcapsule is an anticipated delivery system for protecting probiotics from harsh GI environments and improving survival rates by encapsulating cells in the polymer matrix. Several techniques have been proposed to fulfill effective entrapment including extrusion, spray drying and microfluidics [56,57,58]. Considering that operational processes and conditions have a significant impact on cellular viability and encapsulation efficiency, completing a systematic summary of existing techniques was necessary.

### 3.1. Extrusion

Extrusion is a common technique where probiotics and wall materials are mixed and dropped into hardening fluids to construct probiotic-loaded microparticles [56]. Zhou et al. reported an acid-resistant microcapsule for the entrapment of *Streptococcus thermophilus* IFFI 6038 [59]. Probiotics were protected with the addition of trehalose and alginate, which was added into CaCl_2_ solution dropwise. However, 2 log losses in viability were observed in an acidic environment, indicating a lack of acid stability. Further coating with chitosan showed an enhancement in external environment tolerance. Random probiotic distribution in a polysaccharide matrix was averse to sufficient protection because the marginally distributed probiotics were easily exposed to the external environment. Therefore, a progressive technique based on extrusion is necessary.

**Figure 3:**
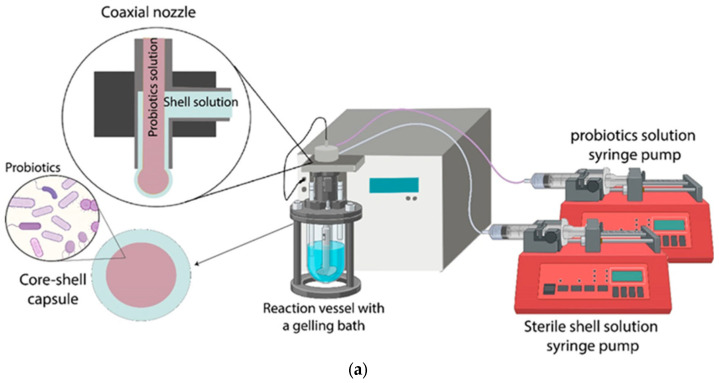

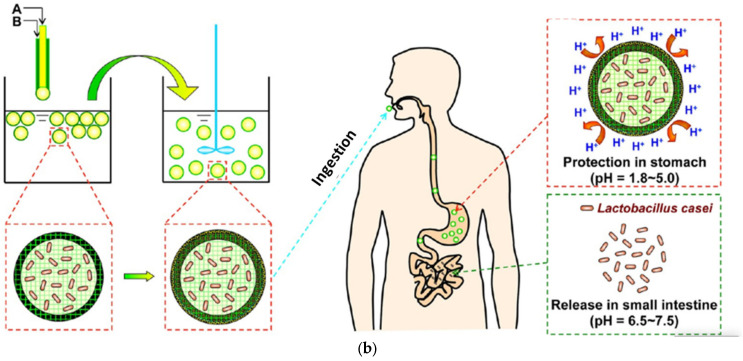
Encapsulation probiotics in core-shell capsule by co-extrusion technique. (**a**) A schematic diagram of co-extrusion equipment [60] and (**b**) pH-responsive probiotic-loaded microcapsules prepared by co-extrusion technique. “A” is probiotics and alginate solution, “B” is pure alginate solution [61].

Co-extrusion has been developed for the construction of core-shell microcapsules to achieve better sheltering of probiotics from external environments [60]. Different from traditional extrusion techniques, co-extrusion involves a coaxial nozzle which pumps a water-in-water jet, where the inner stream is composed of probiotics and the outer stream consists of a shell matrix (Figure 3a). The dual-fluid jet breaks up into small droplets and drips into a hardened solution to form core-shell microcapsules. A prominent superiority of co-extrusion over traditional extrusion is the formation of an outer shell to enclose inner probiotics, demonstrating better gastric acid resistance. Mei et al. developed core-shell microcapsules loaded with *Lactobacillus casei* CICC 23185 by co-extrusion methods [61]. As shown in Figure 3b, the composite shell composed of alginate-Ca^2+^ and protamine demonstrates acid resistance and pH responsiveness. In the simulated gastric environment, alginate-Ca^2+^ presents neutral potential while protamine is positive. Electrostatic repulsion between protamine results in the blockage of the alginate network and outer fluid diffusion is limited. In contrast, protamine is degraded by trypsin in the intestine, resulting in structural fragmentation and drug release.

### 3.2. Emulsion

**Figure 4:**
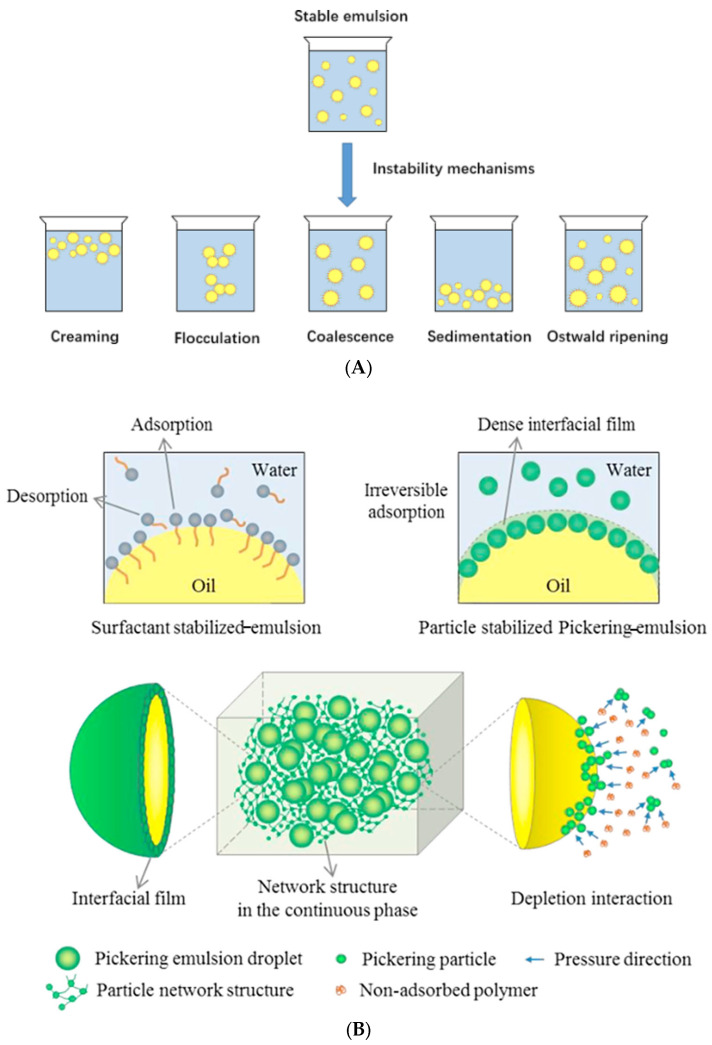
Emulsion instability mechanisms and interfacial stability of a Pickering emulsion. (**A**) Emulsion instability mechanisms caused by creaming, flocculation, coalescence, sedimentation and Ostwald ripening [66]. (**B**) Emulsifier types and interfacial stabilization (surfactant and pickering particle) mechanism of emulsion [67].

Although the extrusion technique was established to protect probiotics from gastrointestinal environments, process scaling restrict its industrial application. Compared with extrusion, emulsion, which is prepared by homogenization or ultrasound, is easy to scale up. In consideration of the water solubility of probiotics, an oil phase or a protective shell is necessary for the protection of inner probiotics solution. Therefore, water-in-oil (*w*/*o*), water-in-oil-in-water (*w*/*o*/*w*) and water-in-water (*w*/*w*) Pickering emulsions are commonly applied for probiotic encapsulation [62,63,64,65] Hydrophilic surfactant whose hydrophobic chains are imbedded in the oil phase while its hydrophilic portions are inserted into the water phase serves as an interfacial stabilizer for *w*/*o* and *w*/*o*/*w* probiotic-encapsulated emulsions. Unsatisfactorily, their deficient stability due to Ostwald ripening, sedimentation, coalescence, flocculation and creaming makes them averse to the storage of probiotics (Figure 4A), for which polysaccharide solid particles are therefore leveraged to form an interfacial layer to stop emulsion droplets from aggregating [66]. As illustrated in Figure 4B, irreversible adsorption of solid particles at droplet interfaces prevents emulsion from coalescing by steric hindrance. Another stability mechanism is owing to the dense interfacial film constructed by aggregated particles which restricts droplet movement. In addition, depletion interaction induces particles to form networks and trap the droplets, preventing droplet collision and aggregation [67].

Zhang et al. reported a *w*/*w* Pickering emulsion, which was stabilized by microcrystalline cellulose (MCC) and hydroxypropyl methyl cellulose as an interfacial stabilizer to encapsulate *Lactobacillus helveticus* CICC 22536 [65]. The stability of the emulsion was proven to be driven by MCC electrostatic repulsion. Xie et al. constructed an interfacial crosslinking *w*/*w* Pickering emulsion loaded with *Lactobacillus helveticus* CICC 22536 to improve its microstructural stability and resilience to gastric acid degradation [68]. In the emulsion system, cellulose crystals were absorbed at the interface to serve as a solid stabilizer, while an interfacial film composed of crosslinked alginates was constructed by the release of Ca^2+^ from CaCO_3_ and electrostatic interaction with alginate sodium at the *w*/*w* interface. An increase of cell viability from 5.200 × 10^4^ CFU/mL (naked cells) to 7.563 × 10^7^ CFU/mL (cells loaded in *w*/*w* Pickering emulsion) indicated that interfacial crosslinking is a potential strategy in oral probiotic delivery. Polysaccharides are not only applied as interfacial films, but also used for coating materials, networks, gelation materials and solid stabilizers [16,62,69]. The applications of polysaccharides in probiotic-loaded emulsions are listed in Table 2.

### 3.3. Spray Drying

**Figure 5:**
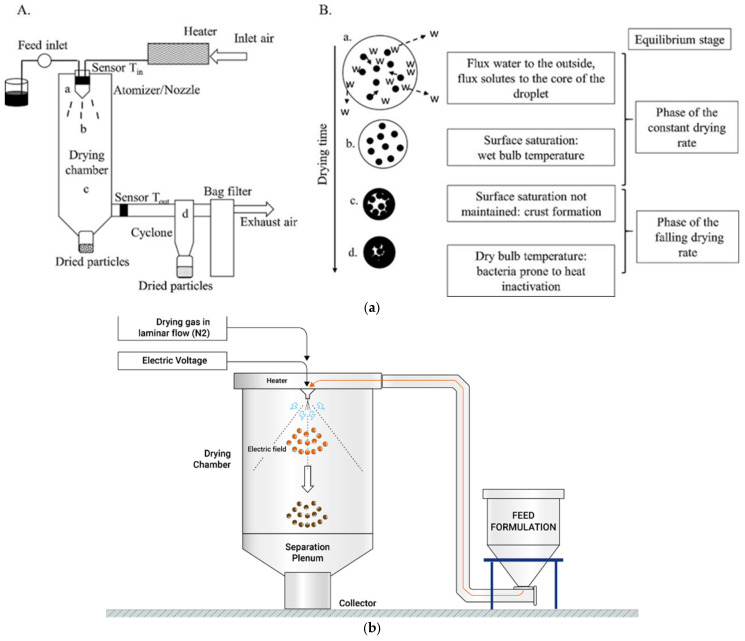

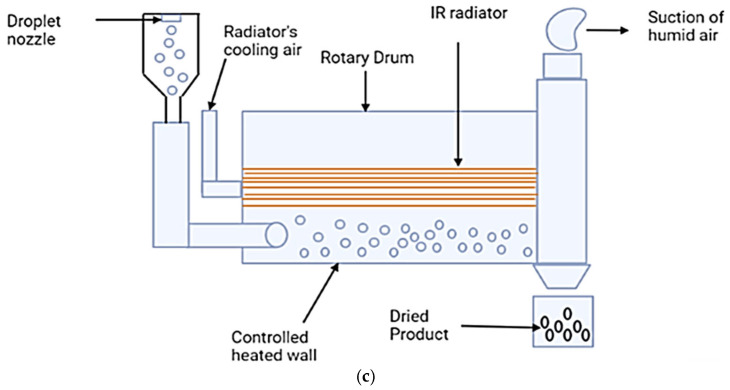
Existing spray drying techniques for microparticle preparation. (**a**) Concurrent spray drying process. Subfigure (**A**) illustrates the technology process of concurrent spray drying process; Subfigure (**B**) demonstrates different stages during drying. [75]. (**b**) Electrostatic spray drying process [76]. (**c**) Spray freeze drying equipment [77].

Their short shelf life and environmental sensitivity are two restrictions of the application of probiotics. Compared with cryopreservation, dehydration is an effective alternative in terms of energy conservation and non-limitation to storage temperature (Perdana et al., 2015). Based on the contact angle of air and fluid, spray drying can be divided into cocurrent, mixed and counter-current flow [75]. For cocurrent spray drying (CSD), hot air and atomized droplets are simultaneously transported into a drying chamber from the top nozzle (Figure 5a). The air presents the highest temperature at the inlet of the drying chamber and gradually cools down during transportation which can prevent microparticles from forming at the bottom and exposing them to a hyperthermic environment. Therefore, CSD has been commonly applied in the preparation of probiotic microparticles to maintain cell viability.

Compared with spray drying, an electric field is introduced into electrostatic spray drying (ESD) to facilitate water removal [76,78]. As shown in Figure 5b, feed liquid is pumped into the drying chamber and atomized into droplets under the action of pressured gas and electric voltage. In general, water introduces a considerable dipole moment into the system, causing it to gather at the edges of droplets under the electrostatic force [79]. As for traditional spray drying, water evaporation occurs at the droplet surface and a solid shell is formed, which restricts the diffusion and volatilization of inner water. Therefore, droplet drying is easier for ESD than that of spray drying, allowing a lower process temperature (about 90 °C). Zaetim et al. reported alginate-chitosan double-layered microcapsules encapsulated with *Lactobacillus plantarum* and *Bifidobacterium lactis* by ESD [80]. The survival rate after microencapsulation was up to 98.1%, indicating that ESD is an appropriate approach for producing microcapsules loaded with high concentrations of probiotics.

Although the operating temperature of ESD is lower than that of spray drying, overheating is still a significant challenge for probiotic dehydration at a heating temperature up to 90 °C. To overcome this obstacle, spray freeze drying (SFD), which is used in frosty conditions, is utilized for bioactive substrates. As shown in Figure 5c, SFD involves three steps including solvent atomization into droplets, heat exchange with a cold stream, and solidified solidification, ice sublimation and microparticle formation [77,81]. The freezing rate is an essential factor determining ice crystal growth, which has an influence on extracellular osmotic pressure and a membrane lipid’s physical state. Semyonov et al. encapsulated *Lactobacillus paracasei* in a saccharide-based matrix to avoid cell damage during the freezing stage [82]. Polysaccharide acts as a protective agent by forming highly viscous glasses, providing shelter from outer ice crystals. Meanwhile, the addition of low-molecular-weight sugar enables double insurance for cellular structural stabilization by forming hydrogen bonds with lipid polar heads [83]. Therefore, the addition of saccharide is of great importance for probiotic micronization by SFD.

### 3.4. Innovative Technologies

Although multiple technologies have been established to prepare probiotic ODSs, the updating and invention of new technology is still necessary for constructing precise and desirable delivery systems.

In recent years, surface decoration has emerged as a promising strategy to generate cell-in-shell structures with functional outer layers as well as environment tolerance. Unlike traditional intricate surface modification methods, Pan et al. presented a facile approach to funtionalizing an *Escherichia coli* Nissle 1917 surface using a co-depositing polymerization method. Polydopamine (PDA) was deposited on the cell surface by forming covalent bonds with nucleophilic moieties of membrane glycoproteins. Then, chitosan was further grafted with PDA to endow the *Escherichia coli* Nissle 1917 with a colon-targeting ability. Chitosan decoration resulted in 15 times more survival in SGF and 5 times higher accumulation at an inflamed colon site. Moreover, this modification approach is easy to realize and can be versatile for various cells [84].

**Figure 6:**
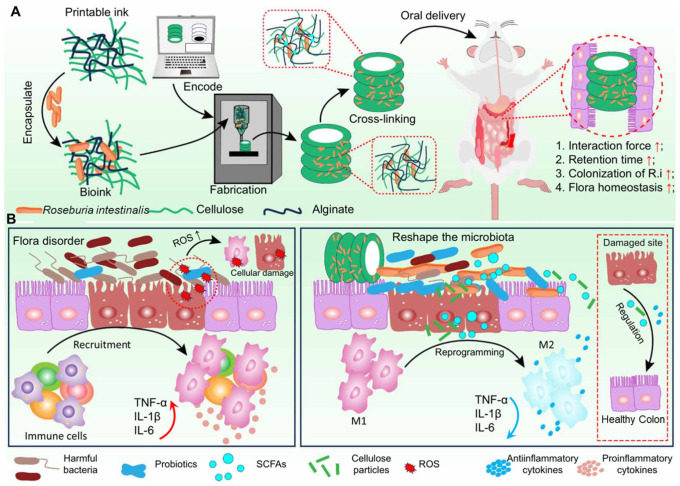
Probiotic ODS constructed by 3D printed technology. (**A**), Preparation process of probiotic-loaded 3D scaffold; (**B**), Intestinal inflammation regulation mechanism [65].

In addition to GI tolerance and colon targeting, retention duration in the colon is another critical factor influencing probiotic action. Updated research has spotlighted the importance of ODS structural design, which will directly affect adhesion to the intestine and retention efficiency. With the development of three-dimensionally (3D) printed technology and its widespread application, probiotic ODSs can be designed with specific structures by 3D printing to adjust for GI transportation behavior. As shown in Figure 6, hydroxy methyl cellulose/sodium alginate was applied as a bioink to form a probiotic-loaded scaffold with a cylinder-like, cube-like and three-prism-like “tube” shape. 3D scaffolding not only increased colon retention duration to 144 h, but also regulated the immune ecosystem as a dietary fiber [65]. Therefore, 3D printing is a prospective platform for constructing shape-controllable probiotic ODSs.

## 4. Challenges and Future Prospects for Probiotic ODSs

Probiotics have been proven to benefit the balance of intestinal microbiota and overall health, for which various ODSs have been developed to achieve oral administration. Although existing probiotic ODSs exhibit superiority in gastrointestinal stability, mucus adhesion and intestine-responsive release profile, there are still some challenges as well as prospects. In this section, key points of probiotic ODS construction and their potential applications will be discussed.

### 4.1. Probiotic Viability Retention During the Dehydration Process

As discussed in Section 3.3, a decrease in cell viability has been a stumbling block for probiotics’ preservation. Probiotics usually experience a dramatic decline in cell viability in their early storage stage, attributed to changes in membrane lipid composition and free radical accumulation [85]. In this case, dehydration is an ideal approach to prolonging the short half-life of probiotics by slowing down their metabolism. However, cell membrane phospholipids tend to rearrange themselves from liquid crystalline to gel phases along with dehydration. The removal of water results in increased van der Waals interactions and lateral compressive stresses, which lead to membrane phase transition. Under the treatment of heat, the gel phase is further oriented into a hexagonal form, causing the membrane bilayer structure to be destroyed. Another reason for probiotic death induced by dehydration is the inactivation of embedded membrane protein. The movement of protein is closely associated with membrane fluidity, which is restricted at a low water concentration [86].

Saccharides such as trehalose are applied as a protectant to avoid dehydration-related cell death. Sugars readily form hydrogen bonds with the polar heads of the lipid bilayer, replacing water molecules. Therefore, the abovementioned cell membrane phase transition due to water removal can be avoided, prompting enhanced probiotic survival during drying [87]. Unlike the molecular interaction between saccharides and phospholipids, the rigid structure of polysaccharides is averse to intermolecular actions. Advantageously, polysaccharides are reported to prevent excessive dehydration and protect membrane protein activity, which is a breakthrough for maintaining the probiotic survival rate during the drying process [88,89].

### 4.2. Core-Shell Probiotic Microparticle Based on a Polyelectrolyte Complex

Employing a core-shell structure appears to be a predominant strategy for enclosing acid-sensitive probiotics within a protective shell. A polyelectrolyte complex (PEC) demonstrates advantages in acid stability, pH responsiveness biodegradability and safety, and it is considered as a potential layer for ODSs [90]. Even though extrusion and emulsion have been employed for constructing core-shell structures based on PECs, the obtained microparticles in the wet state are averse to the storage of probiotics. Spray drying is commonly applied for probiotic micronization; however, a water-soluble matrix of prepared microparticles is limited in its gastrointestinal resistance. Therefore, dehydration microparticles with a polyelectrolyte complex shell and the combination of existing technology are of great necessary.

Tan et al. developed a modified spray drying technique to combine particle formation, in situ crosslinking and dehydration for the first time [57]. Aqueous alginates/probiotics were pumped into an inner feeding pipe while a crosslinking agent was used in the outer channel. In situ crosslinking was achieved by the collision of two phases through a coaxial nozzle, and dried microparticles were obtained via water evaporation under the treatment of hot nitrogen. However, an operating temperature of up to 140 °C poses the risk of protein denaturation and cell inactivation. As a consequence, research on the optimization of in situ crosslinking spray drying and the development of new technology is still needed.

## 5. Conclusions

Probiotics are not only important nutritional supplements for maintaining balance in gut microbiota and immune regulation, but also have potential therapeutic effects. The main constraint of the oral administration of probiotics is gastrointestinal instability and a short half-life. This review summarized the applications of polysaccharides and crosslinking methods in ODS protective shells to avoid probiotic inactivation during GI transportation. Extrusion and emulsion have been devoted to preparing probiotic ODSs with a crosslinking layer based on polysaccharides while further dehydration is required for probiotic preservation. Although various spray drying techniques including CSD, ESD and SFD have been developed for probiotic micronization, the design of probiotic microparticles with a polyelectrolyte complex shell is still challenging. Further research and exploration are still imperative.

## Figures and Tables

**Table 1:** Cellulose and cellulose derivates applied in probiotic encapsulation.

Cellulose/ Cellulose Derivate	Probiotics	Encapsulation System	Characterization	Superiority	Reference
CMC-Na	*Lactobacillus reuteri* KUB-AC5	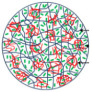 Microcapsule	159.92 ± 3.96 μm; Nearly spherical microcapsules	(1) Encapsulation efficiency reached up to 91.78 ± 0.03%. (2) Heat survival was improved by the protection of polysaccharide microcapsules.	[33]
Cellulose acetate	*Escherichia coli* Nissle 1917	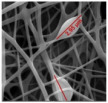 Hybrid fiber	754 ± 497 nm; Uniform fibers	(1) Cell viability was maintained during the process of electrospinning. (2) Probiotic survivability in the gastrointestinal environment was significantly improved under the action of composed fibers.	[26]
Cellulose	*Lactobacillus plantarum* CICC 6240	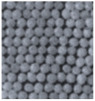 Microgel	500 μm; Core-shell spherical microgels	(1) Probiotics were distributed in the core of microgels and the shell sheltered *L. plantarum* from acid gastric fluids. (2) Controlled release of probiotics lasting for 360 min was achieved in intestinal environment.	[34]
Nanocrystal cellulose	*Lactobacillus helveticus* CICC 22536	*** 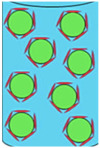 ****w*/*w* emulsion	5–6 μm	(1) Long-term stability of *w/w* emulsion was achieved with the addition of nanocrystal cellulose. (2) Probiotics were encapsulated in the emulsion at a relatively high density of 5.46 × 10^9^–6.59 × 10^9^ CFU/mL.	[35])
Cellulose sulphate	*Lactobacillus acidophilus* DMS 20079, *Lactobacillus johnsonii* DMS 10533, *Lactobacillus casei* DSM 20011 *and Bifidobacterium longum subsp. infantis (B. infantis)* DMS 20088	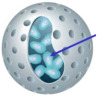 Porous capsule	0.7 mm	(1) The viability of probiotics in porous capsules was satisfactory compared to that of uncoated probiotics following exposure to stomach fluids. (2) Effective protection during gastrointestinal and colon-specific delivery by cellulose sulfate-based capsules was confirmed in a mouse model.	[29]

**Table 2:** The applications of polysaccharides in improving emulsion stability.

Polysaccharide	Probiotic	Emulsion Type	Stability Mechanism andAdvantages
Soy hull polysaccharide	*Lactobacillus plantarum*	*o*/*w*	(1) Soy hull polysaccharide (SHP) interacted with soy protein isolate (SPI) by electrostatic interaction and hydrogen bonding. The complex was a semi-crystalline structure and provided a shield for probiotics. (2) Emulsion remained stable in acid stomach fluid due to protonation of polar residues, prompting a stronger interaction for SHP/SPI. (3) A colon-specific drug release profile was confirmed in vitro [64].
Apple pectin/alginate	*Lactobacillus reuteri*	*w*/*o*/*w*	(1) Apple pectin-zein nanoparticles served as emulsifiers, protecting the emulsion from aggregation by electrostatic repulsion. (2) Alginate crosslinked gel enclosed emulsion droplets and provided a protective shell. (3) Survival rate of probiotics reached up to 75.11% after simulated gastrointestinal digestion [70].
κCarrageenan/carboxymethyl chitosan/alginate	*Lactobacillus rhamnosus*	*w*/*o*/*w*	(1) κCarrageenan-sodium caseinate nanoparticles were obtained by Maillard reaction and added to the emulsion to decrease interfacial tension. (2) Carboxymethyl chitosan and sodium alginate were theorized to have tightly bonded in a gastric environment due to the electrostatic attraction of –COO^−^ and –NH3^+^ while becoming loose in basic intestinal fluids, presenting pH-responsive drug release profiles [63].
Alginate/carboxymethyl konjac glucomannan/chitosan	*Lactobacillus reuteri*	*w*/*o*/*w*	(1) Nanogels were prepared by the amidation reaction of carboxymethyl konjac glucomannan and chitosan (CMKGM-CS), which was able to be absorbed at the oil/water interface to ameliorate emulsion stability. (2) A network of alginate hydrogels embedded with a *w*/*o*/*w* emulsion was constructed to provide shelter from acid gastric fluids while becoming loose in intestinal fluids, achieving a targeted drug release. (3) Emulsion was stable after incubation at 4 °C for 90 days or 25 °C for 63 days [71].
Chitosan/alginate	*Lactobacillus plantarum*	*w*/*o*/*w*	(1) Probiotics were encapsulated in the inner water phase of *w*/*o*/*w*, which were coated with multilayers based on polysaccharides. (2) Coating layers were established by the electrostatic crosslinking of anionic polysaccharides with cationic ones or a crosslinking agent. (3) Multilayers exhibited better protection of probiotics than single layers, and cell viability after digestion was increased from 31.6% (single layer) to 75% (three coating layers) [72].
Fucoidan/chitosan	*Lactobacillus* *bulgaricus*	*w*/*o*/*w*	(1) Fucoidan was modified with carboxyl groups to interact with amino groups of chitosan and form nanogels. (2) Nanogels were confirmed to anchor and cover the oil/water interface to stabilize emulsion droplets. (3) The *w*/*o*/*w* emulsion was incorporated in alginate hydrogel particles, which exhibited a pH-responsive swelling capacity, providing potential for oral probiotic delivery [73].
Hydroxypropyl methylcellulose (HPMC)/dextran	*Lactobacillus plantarum*	*w*/*w* Pickering emulsion	(1) Cellulose crystal acted as a solid stabilizer for the *w*/*w* Pickering emulsion whose concentration had an obvious effect on the emulsion’s stability. (2) An increase in HPMC was beneficial to the decrease in emulsion diameters, owing to the increased emulsion viscosity and restricted emulsion mobility. (3) The preservation duration was up to 90 days and cell viability after gastrointestinal digestion remained at 3.38 × 10^6^ CFU/g, demonstrating that the *w*/*w* Pickering emulsion stabilized by HPMC was a promising strategy for oral probiotic delivery [74].

## Data Availability

All data are contained within this article.
